# Simple Preparation and Characterization of Hierarchical Flower-like NiCo_2_O_4_ Nanoplates: Applications for Sunset Yellow Electrochemical Analysis

**DOI:** 10.3390/bios12110912

**Published:** 2022-10-22

**Authors:** Hadi Beitollahi, Somayeh Tajik, Zahra Dourandish, Fariba Garkani Nejad

**Affiliations:** 1 Environment Department, Institute of Science and High Technology and Environmental Sciences, Graduate University of Advanced Technology, Kerman P.O. Box 76318-85356, Iran; 2 Research Center of Tropical and Infectious Diseases, Kerman University of Medical Sciences, Kerman P.O. Box 76169-13555, Iran

**Keywords:** sunset yellow, flower-like NiCo_2_O_4_ nanoplates, voltammetry, modified electrode

## Abstract

The current work was performed to construct a novel electrochemical sensing system for determination of sunset yellow via the modification of screen-printed graphite electrode modified with hierarchical flower-like NiCo_2_O_4_ nanoplates (NiCo_2_O_4_/SPGE). The prepared material (hierarchical flower-like NiCo_2_O_4_ nanoplates) was analyzed by diverse microscopic and spectroscopic approaches for the crystallinity, composition, and morphology. Chronoamperometry, differential pulse voltammetry, linear sweep voltammetry, and cyclic voltammetry were used for determination of the electrochemical behavior of sunset yellow. The as-fabricated sensor had appreciable electro-catalytic performance and current sensitivity in detecting the sunset yellow. There were some advantages for NiCo_2_O_4_/SPGE under the optimized circumstances of sunset yellow determination, including a broad dynamic linear between 0.02 and 145.0 µM, high sensitivity of 0.67 μA/(μM.cm^2^), and a narrow limit of detection of 0.008 μM. The practical applicability of the proposed sensor was verified by determining the sunset yellow in real matrices, with satisfactory recoveries.

## 1. Introduction

Food additives applied in processed foodstuff can normally improve some features such as flavor, appearance, color, taste, nutritive value, preservation, and texture [1]. Color has always encouraged consumers to buy a food product, usually because of the mere fact that it is visual at the first sight. A buyer can be affected negatively or positively by colors. In the food industry, natural dyes in food products can be intensified or preserved using food dyes. The pharmaceutical industry also exploits edible coloring to create a different look in their products. The edible colors can be natural or synthetic. Natural dyes originate from animal or plant sources, by extraction through physical techniques such as riboflavin. The natural color of the edible products may be lost during various stages including heating, processing, storage, and distribution. Synthetic dyes are composed of artificially synthesized organic and inorganic compounds. Among these, a special place in food industry has been established for the synthetic colorants as good alternatives to natural dyes because of appreciable merits such as long-lasting stability, easy coloring, minimal microbiological and chemical or physical contamination [2,3]. Azo dyes with a broad spectrum of colors owing to a unique chemical structure account for about 65% of the commercial dye market around the world [4,5]. Disodium 2-hydroxy-1-(4-sulphonatophenylazo) naphthalene-6-sulphonate, or sunset yellow, as a widely used synthetic azo dye can be found in various beverages, foods, medicines, colorings, and cosmetics, which is cost-effective with stable structure and bright color [6,7,8]. The allowance limit of sunset yellow as food additive must be ˂50 ppm [9]. Hence, doses exceeding this limit can be associated with complications such as kidney failures, attention deficit hyperactivity disorder (ADHD), hepatocellular damage, cancers, and headache [10,11]. Accordingly, there have been various analytical approaches for sensing and quantifying the sunset yellow in food matrices, some of which are coupling ionic liquid-based aqueous two-phase systems (IL-ATPSs) with high performance liquid chromatography (HPLC) [12], reversed-phase ion pair high-performance liquid chromatography [13], spectrophotometry [14], HPLC/mass spectrometry (HPLC–MS) [15], ion-pair liquid chromatography with on-line photodiode-array and electrospray mass spectrometry [16], capillary electrophoresis [17], and high-performance thin-layer chromatographic combined with image processing of scanned chromatograms [18]. However, their use is often limited due to reasons such as being expensive, time-consuming, or requiring tedious pretreatment.

Among these, electrochemical analysis has had special place in determining the bioactive molecules, nutrients, drugs, contaminants, and food additives owing to cost-effectiveness, facile use, rapidity, high sensitivity and selectivity [19,20,21,22,23,24,25,26,27,28,29,30,31,32,33], thereby making them a good alternative to the above-mentioned techniques. There are several conventional solids (such as glassy carbon, carbon paste, and gold) or disposable electrodes (such as screen-printed electrodes or SPEs) to construct the electrochemical sensors. Special attention in the field of electroanalytical research has recently been drawn to SPEs in the manufacture of (bio) sensors. The SPEs are appropriate electrochemical transducers because of high sensitivity, ease of use, and cost-effective properties when comparing with conventional diagnostics [34,35,36,37,38]. Such devices possess a miniaturized system consisting of the working, auxiliary, and reference electrodes. Hence, there is a need for the small volume of solution for electrochemical determination, which is indeed interesting in terms of green chemistry [39,40]. Chemically modified electrodes (CMEs) are the result of the intentional fixation of a modifying agent on the surface of electrode by various physical and chemical methods [41,42]. Many studies have shown that improvements in the sensitivity and selectivity of electrochemical sensors are achieved due to modifications of the electrode surface [43,44]. Nanomaterial-supported electrochemical sensing systems have been considered by many researchers due to their capability for carrying out the electrochemical analysis of diverse analytes [45]. The electrode surface modification using diverse nanostructures can enhance the analyte-specific electrochemical reactivity and sensitivity [46,47].

Nanomaterials and their applications in various fields have become a distinct and active area of scientific and technological developments over the recent years [48,49,50,51,52,53,54,55,56]. In this regard, the metal oxide nanoparticles are applied extensively in some fields such as energy production, food technology and preservative, medicine, catalysis and electrocatalysis because of intrinsic redox features and morphological and structural flexibility [57,58,59,60,61]. One of the best electrode modifiers is binary transition-metal oxide because of superior resistance to deactivation, appreciable selectivity, and high catalytic performance [62,63]; for example, NiCo_2_O_4_ nanostructures are mixed-metal oxides successful for sensors owing to electrochemical catalytic performance, high biocompatibility, commendable electronic conductivity, non-toxicity, and low cost. NiCo_2_O_4_ has greater electronic conductivity and electrochemical performance when comparing with cobalt and nickel oxides. The synergic impact of nickel and cobalt elements in NiCo_2_O_4_ provides a richer diversity of redox reactions when comparing with the monometallic NiO and Co_3_O_4_ [64,65,66]. Accordingly, NiCo_2_O_4_ is considered as an electrochemical material but still suffers from some problems with improvement in a small surface area, pore size, and intrinsically poor electro-conductivity [67]. Such bottlenecks can be circumvented by using materials based on two-dimensional or sheet-like morphology owing to the merits associated with large pore size, high surface area, short paths, and fast reaction kinetics for redox process [68,69]. Thus, 2D or sheet-like NiCo_2_O_4_ can serve as an electrode material for the fabrication of electrochemical sensors.

The current work was performed to fabricate hierarchical flower-like NiCo_2_O_4_ nanoplates by a facile method and to characterize their structure and morphology by diverse techniques. Then, the as-produced nanoplates were used to modify the SPGE surface to construct a new sunset yellow sensor (NiCo_2_O_4_/SPGE). The resulting modified electrode (NiCo_2_O_4_/SPGE) had a great sensitivity towards the sunset yellow, possessing a narrow limit of detection (LOD) and a broad linear range. The analytical performance and practical applicability of proposed sensor was determined by sensing sunset yellow in real food specimens.

## 2. Experimental

### 2.1. Equipments

A Metrohm Autolab PGSTAT 320N Potentiostat/Galvanostat Analyzer (Herisau, Switzerland) with GPES (General Purpose Electrochemical System-version 4.9) software was applied for all electrochemical determinations at ambient temperature. Cyclic voltammetry (CV), Chronoamperometry (CA), differential pulse voltammetry (DPV) and linear sweep voltammetry (LSV) were employed to characterize the electro-analytical performance of the modified electrode toward sunset yellow. The electrochemical sensors were prepared by DRP-110 SPEs (DropSens, Oviedo, Spain) including a silver pseudo-reference electrode, graphite working electrode, and graphite auxiliary electrode. A Metrohm 713 pH-meter with glass electrode (Metrohm AG, Herisau, Switzerland) was used to determine and adjust the pH of the solution.

A Panalytical X’Pert Pro X-ray diffractometer (Almelo, The Netherlands) applying a Cu/Kα radiation (λ:1.54 Å) was used for X-ray diffraction (XRD) analysis and a Bruker Tensor II spectrometer (Bruker, Karlsruhe, Germany) was employed to capture the Fourier transform-infrared (FT-IR) spectra. A MIRA3 scanning electron microscope (Tescan, Brno, Czech Republic) coupled with an X-ray spectroscopy (EDS) detector was utilized for field emission-scanning electron microscopy (FE-SEM) images and elemental analysis.

### 2.2. Solvents and Chemicals

All solvents and chemicals applied in our protocol had analytical grade belonging to Merck and Sigma-Aldrich. Phosphate buffer solution (PBS) was prepared by phosphoric acid and adjusted by NaOH to the desired pH value.

### 2.3. Preparation of Hierarchical Flower-like NiCo_2_O_4_ Nanoplates

The protocol proposed by Chu et al., with slight modification, was followed to construct hierarchical flower-like NiCo_2_O_4_ nanoplates [70]. Thus, Ni(NO_3_)_2_6H_2_O (0.5 mmol, 0.145 gr), Co(NO_3_)_2_6H_2_O (1 mmol, 0.291 gr), NH_4_F (3 mmol, 0.111 gr), and urea (7.5 mmol, 0.45 gr) were dispersed in deionized water (40 mL) while stirring for 40 min until reaching a clear pink solution, followed by placing in a Teflon-lined stainless steel autoclave for three hours at 120 °C. After cooling down to the laboratory temperature, the collected precipitate was rinsed thoroughly with deionized water and oven-dried for 12 h at 65 °C. At last, the obtained product was annealed at 350 °C for 150 min.

### 2.4. Preparation of the NiCo_2_O_4_/SPGE Sensor

**Scheme 1:**
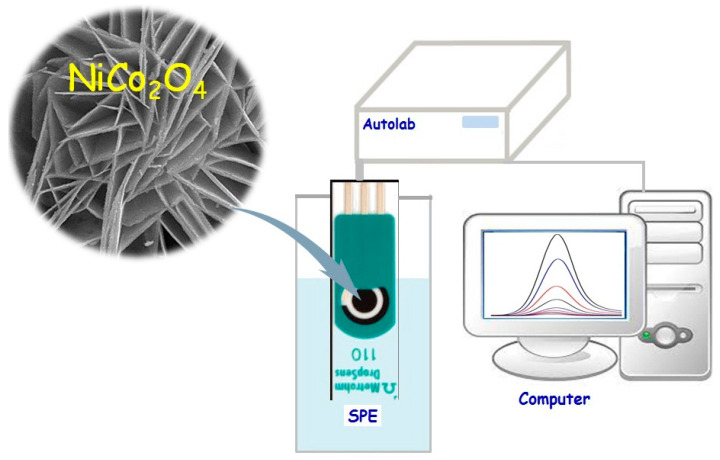
Schematic illustration of NiCo_2_O_4_/SPGE-based sunset yellow electrochemical detection.

A drop-casting technique was followed to fabricate the NiCo_2_O_4_/SPGE (Scheme 1). Thus, a certain amount of as-prepared flower-like NiCo_2_O_4_ nanoplates (1 mg) was subsequently dispersed in deionized water (1 mL) under 20-min ultra-sonication. Then, the well-dispersed suspension (5 µL) was coated on the SPGE surface in a dropwise manner and dried at the laboratory temperature. The electrochemical surface areas of the NiCo_2_O_4_/SPGE and the SPGE were obtained by CV using 1 mM K_3_Fe(CN)_6_ at various scan rates. The electro-active surface area of the modified electrode and un-modified electrode were evaluated by using the Randles–Sevcik equation. The calculated electro-chemical active surface area is 0.15 cm^2^, for the NiCo_2_O_4_/SPGE and 0.02 cm^2^ for the bare SPGE.

### 2.5. Real Sample Preparation

Orange juice and apple juice were purchased from a local market. The fruit juice samples were centrifuged for 40 min at 400 rpm and then filtered. The obtained juice was diluted by PBS (pH = 7.0) and then used for real sample analysis. Moreover, the tap water specimens were filtered prior to analysis using the standard addition method.

## 3. Results and Discussion

### 3.1. Characterization of Hierarchical Flower-like NiCo_2_O_4_ Nanoplates

**Figure 1:**
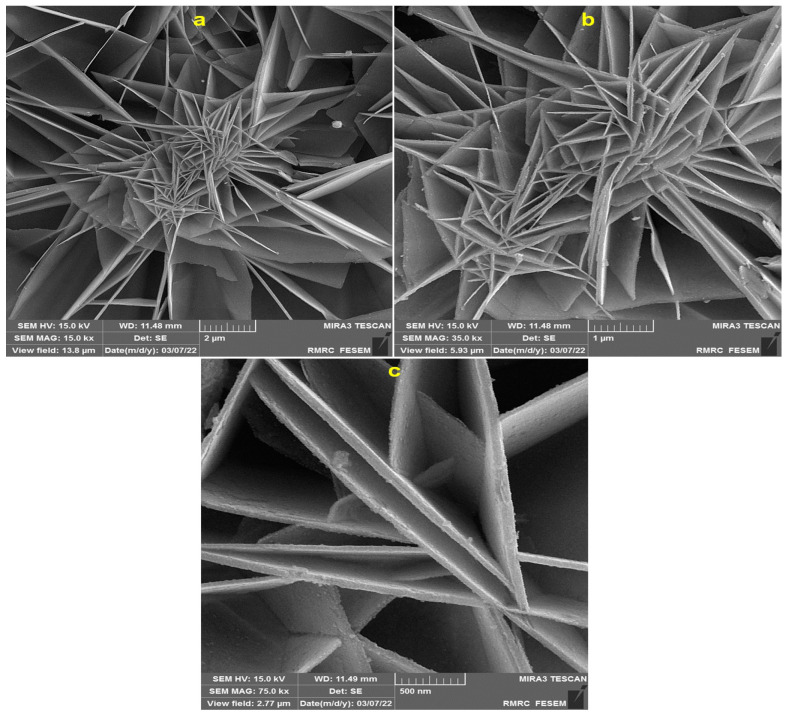
FE-SEM images at diverse magnifications (**a** = 2 µm, **b** = 1 µm and **c** = 500 nm) for hierarchical flower-like NiCo_2_O_4_ nanoplates.

The hierarchical flower-like NiCo_2_O_4_ nanoplates were explored for structure and morphology using FE-SEM images (Figure 1). The self-assembly of 2D NiCo_2_O_4_ nanoplates around a center resulted in the formation of hierarchical flower-like NiCo_2_O_4_ nanostructures. The FE-SEM image at high magnification shows the thickness of ˂60 nm for the produced nanoplates.

**Figure 2:**
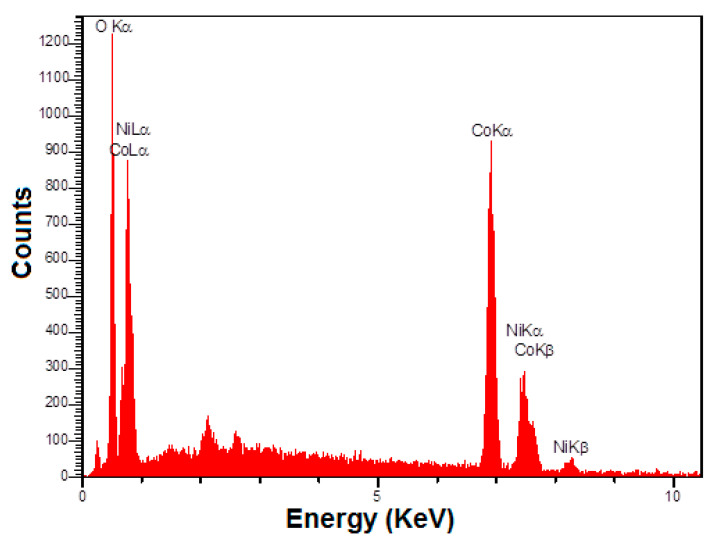
EDS spectrum of hierarchical flower-like NiCo_2_O_4_ nanoplates.

Figure 2 shows the EDS analysis in determining the chemical composition of the hierarchical flower-like NiCo_2_O_4_ nanoplates, the findings of which display Ni, Co, and O elements present in the structure, with no impurity.

**Figure 3:**
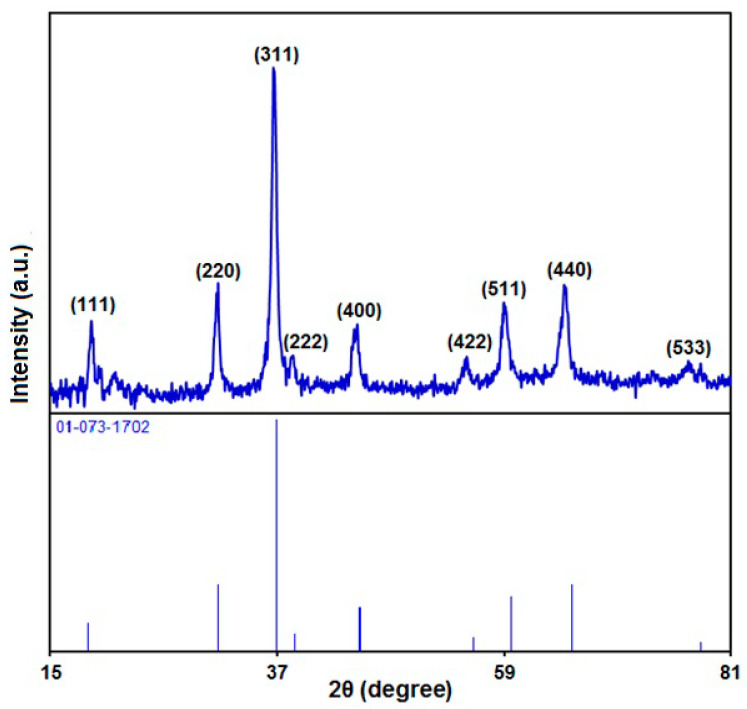
XRD pattern of hierarchical flower-like NiCo_2_O_4_ nanoplates.

Figure 3 illustrates the XRD pattern to explore the crystal phase of hierarchical flower-like NiCo_2_O_4_ nanoplates. The diffraction peaks at 2θ values (indexed to related plane) of 18.9° (111), 31.1° (220), 36.6° (311), 38.5° (222), 44.5° (400), 55.2° (422), 59.0° (511), 64.9° (440), and 77.1° (533) corresponded to the JCPDS standard pattern of NiCo_2_O_4_ (No. 01-073-1702). The distinct crystalline nature is evident based on the intense and sharp diffraction peaks. The absence of extraneous peaks can confirm great purity of as-fabricated NiCo_2_O_4_.

**Figure 4:**
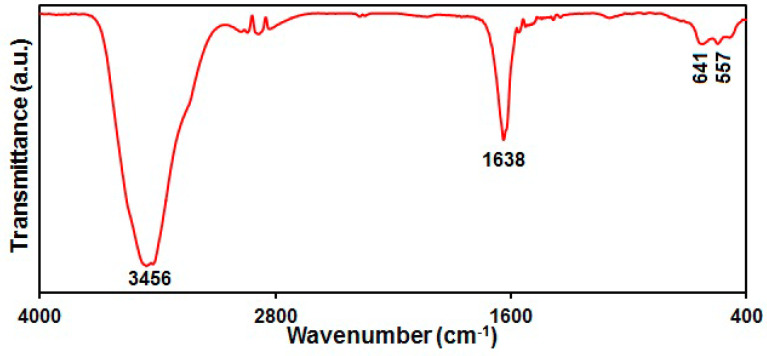
FT-IR spectrum of hierarchical flower-like NiCo_2_O_4_ nanoplates.

The production of NiCo_2_O_4_ was further confirmed via FT-IR spectrum (Figure 4). As seen, the peaks at 3456 and 1638 cm^−1^ were related to the characteristic hydroxyl stretching and bending vibrations in the adsorbed H_2_O [71]. The peaks at 557 and 641 cm^−1^ corresponded to metal–oxygen bond vibrations in the NiCo_2_O_4_ [72].

### 3.2. Electrochemical Characteristics of the Modified Electrode

**Figure 5:**
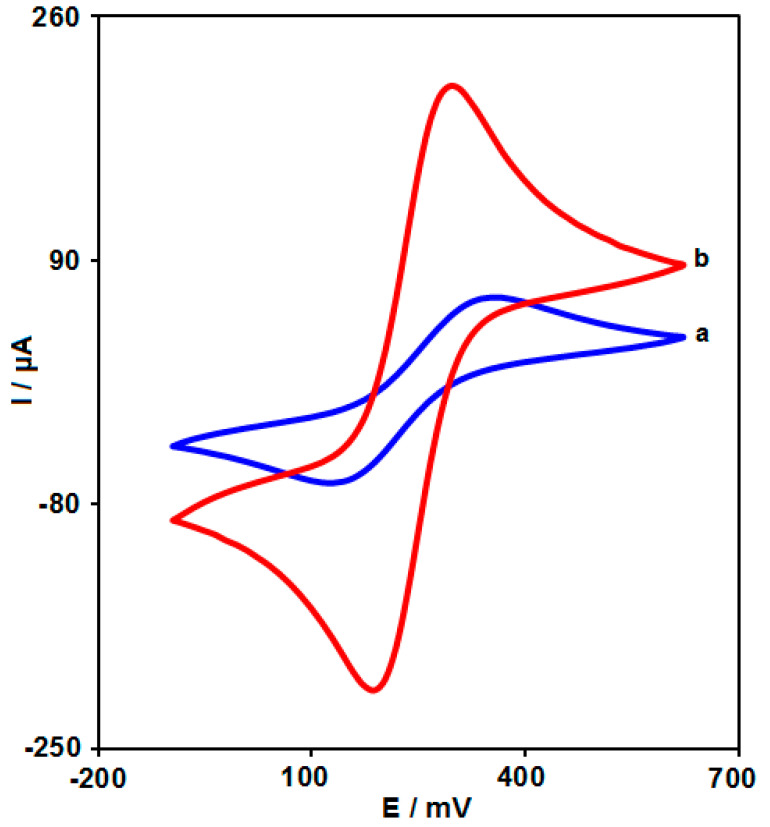
CVs curves of different electrode in PBS (0.1 M, pH 7.0) solution containing 1.0 M KCl and 5.0 mM Fe(CN)6 ^3−/4−^; (a) bare SPGE and (b) NiCo_2_O_4_/SPGE. Scan rate: 100 mV s^−1^.

The electrochemical properties of the modified electrodes were studied by CV in the presence of [Fe(CN)_6_]^3−/4−^ (Figure 5). Obviously, a pair of well-shaped and symmetrical redox peaks occurred at all electrodes, demonstrating that the redox behavior of Fe^III^/Fe^II^ corresponded to a quasi-reversible process. At bare SPGE, a pair of relative weak redox peaks (Ipa = 64 μA; Ipc = −64 μA) appeared at 0.35 and 0.115 V, respectively. After modification of SPGE by NiCo_2_O_4_ nanoplates, the Ipa and Ipc increased to 209 and −209 μA while the peak separation (ΔEp) decreased from 0.235 to 0.105 V.

### 3.3. Influence of pH on Electrochemical Behavior of Sunset Yellow

**Figure 6:**
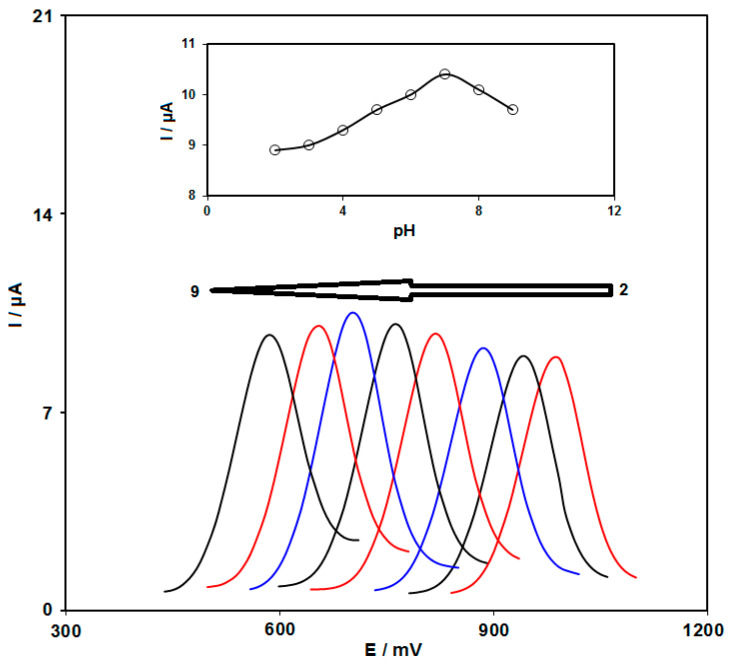
DPVs captured for the oxidation of 100.0 μM sunset yellow contents on the NiCo_2_O_4_/SPGE at variable pH (2.0, 3.0, 4.0, 5.0, 6.0, 7.0, 8.0 and 9.0). Inset: Variation peak current as a function of solution pH.

**Scheme 2:**
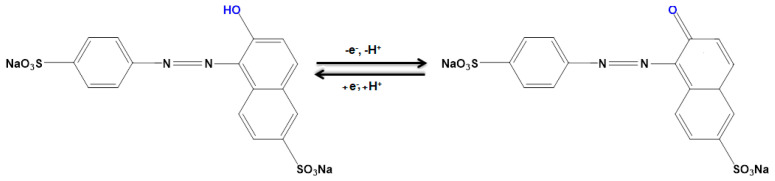
The mechanism of the electrochemical reaction of sunset yellow on the NiCo_2_O_4_/SPGE.

The electrochemical response of sunset yellow in the 0.1 M PBS adjusted to variable pH values (2.0 to 9.0) was explored to determine the influence of the electrolyte solution pH (Figure 6). The results showed that the peak current of sunset yellow oxidation depended on the pH value, so that it reached a maximum with increasing pH up to 7.0 and then decreased with further pH values. Hence, the pH value of 7.0 was considered to be the optimum for subsequent electrochemical determinations (Figure 6, inset). Since the equal amount of proton and electron participates in the redox process, the electrochemical reaction of sunset yellow is 1 electron and 1 proton process. Hence, the electrochemical mechanism of sunset yellow on the NiCo_2_O_4_/SPGE can be inferred from Scheme 2.

### 3.4. Electrochemical Response of Sunset Yellow at Various Electrodes

**Figure 7:**
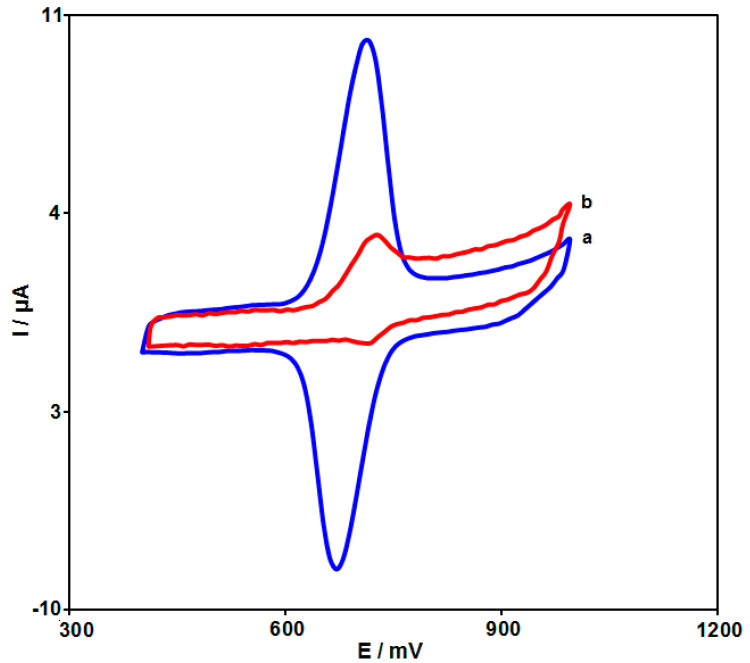
CVs captured for electrochemical reaction of sunset yellow (100.0 μM) in PBS (0.1 M; pH = 7.0) on (a) NiCo_2_O_4_/SPGE and (b) unmodified SPGE with the scan rate of 50 mV/s.

The CVs were captured for the sunset yellow (100.0 μM) electrochemical reaction on the bare SPGE and NiCo_2_O_4_-modified SPGE to explore the electrocatalytic performance of the hierarchical flower-like NiCo_2_O_4_ nanoplates (Figure 7). Figure 7 illustrates the weak oxidation peak on the bare SPGE (Ipa = 3.1 μA), whereas NiCo_2_O_4_-modified SPGE had a significant improvement in the current (Ipa = 10.5 μA). This significant improvement in the oxidation peak can appear because of the appreciable catalytic impact of hierarchical flower-like NiCo_2_O_4_ nanoplates for the sunset yellow electrochemical reaction.

### 3.5. Effect of Scan Rate

**Figure 8:**
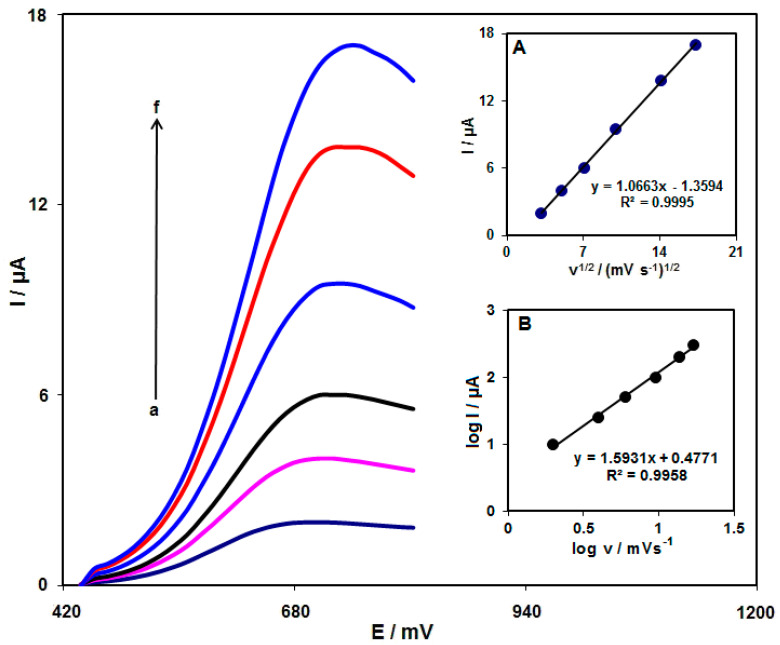
LSVs captured for the oxidation of sunset yellow (60.0 μM) on the NiCo_2_O_4_/SPGE under variable scan rates (a: 10 mV/s, b: 25 mV/s, c: 50 mV/s, d: 100 mV/s, e: 200 mV/s, and f: 300 mV/s). Insets: (**A**) the correlation of Ipa with ν^1/2^. (**B**) Variation of logarithm of current as a function of variation in the logarithm of scan rate.

The LSVs were captured for the oxidation of sunset yellow (60.0 μM) on the NiCo_2_O_4_/SPGE under variable scan rates (Figure 8). There was an apparent gradual elevation in the oxidation peak when the scan rate ranged from 10 to 300 mV/s. As seen in Figure 8 (Inset), the anodic peak current (Ipa) had a linear association with the square root of the scan rate (ν^1/2^). The regression equation was obtained to be Ipa (µA) = 1.0663 ν^1/2^ (mV s^−1^)^1/2^–1.3594 (R^2^ = 0.9995), meaning a controlled diffusion process of the sunset yellow oxidation on the NiCo_2_O_4_/SPGE (Figure 8, Inset A). In addition, the variation of the logarithm of the current as a function of the variation in the logarithm of the scan rate showed a linear behavior that showed the controlled diffusion process of the sunset yellow oxidation on the NiCo_2_O_4_/SPGE (Figure 8, Inset B).

**Figure 9:**
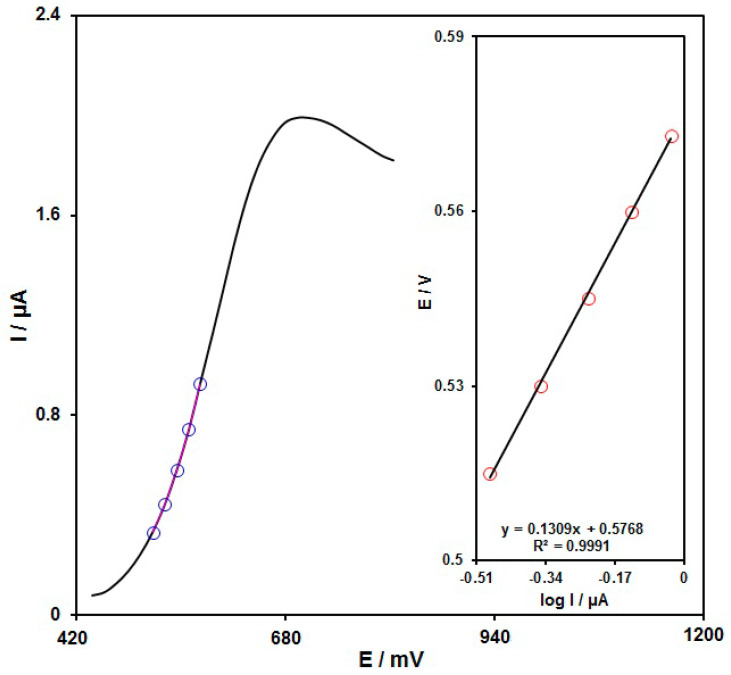
LSV for sunset yellow (60.0 μM) at the scan rate of 10 mV/s. Inset: The Tafel plot from the rising domain of the respective voltammogram.

A Tafel plot (Figure 9 (Inset)) was achieved on the basis of data related to the rising domain of the current–voltage curve at a low scan rate (10 mV/s) for sunset yellow (60.0 μM) to explore the rate-determining step. The linearity of E vs. log I plot clarifies the involvement of electrode process kinetics. The slope from this plot could present the count of transferred electrons during the rate-determining step. Based on Figure 9 (inset), the Tafel slope was estimated to be 0.1309 V for the linear domain of the plot. The slope of the Tafel plot was equal to 2.3RT/n(1−α)F, and the Tafel slope based on Figure 9 (inset) was estimated to be 0.1309 V for the linear domain of the plot. The Tafel slope value reveals that the rate-limiting step is the one-electron transfer process, considering a transfer coefficient (α) of 0.55.

### 3.6. Chronoamperometric Analysis

**Figure 10:**
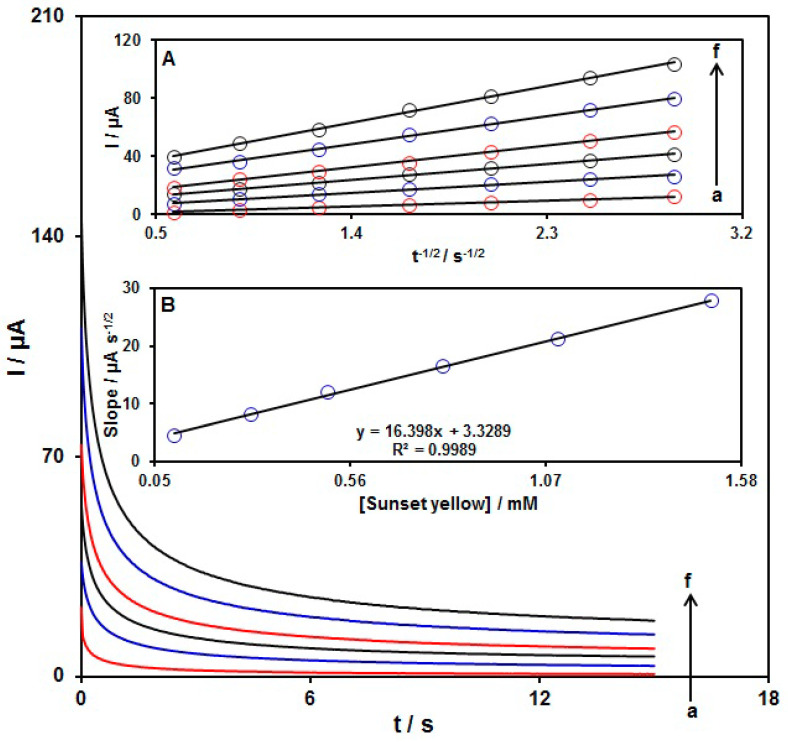
Chronoamperometric behavior of NiCo_2_O_4_/SPGE in PBS (0.1 M; pH = 7.0) at potential of 740 mV for variable sunset yellow contents (a: 0.1 mM, b: 0.3 mM, c: 0.5 mM, d: 0.8 mM, e: 1.1 mM and f: 1.5 mM). Insets: (**A**) Plots of I vs. t^−1/2^ and (**B**) plots of the slopes from the straight lines vs. sunset yellow level.

Chronoamperometry was used to explore the sunset yellow catalytic oxidation on the NiCo_2_O_4_/SPGE surface. Chronoamperometric analysis was done for variable sunset yellow contents on NiCo_2_O_4_/SPGE at the working electrode potential of 740 mV. The chronoamperograms captured for variable sunset yellow contents on the NiCo_2_O_4_/SPGE can be seen in Figure 10. Cottrell’s equation explains the current (I) for electrochemical reaction of an electroactive material with a D value (diffusion coefficient) under a mass transport-limited condition. Figure 10A shows a linear relationship of the I value with t^−12^ for the oxidation of variable sunset yellow contents. The slopes from the obtained straight lines were plotted against variable sunset yellow contents (Figure 10B). The plotted slope and Cottrell equation (*I = nFAD^1/2^C_b_π^−1/2^t^−1/2^*) estimated the D value to be 9.2 × 10^−5^ cm^2^/s for sunset yellow. The mean D value in this work is comparable with the results published in other papers [73,74].

### 3.7. DPV Analysis of Sunset Yellow

**Figure 11:**
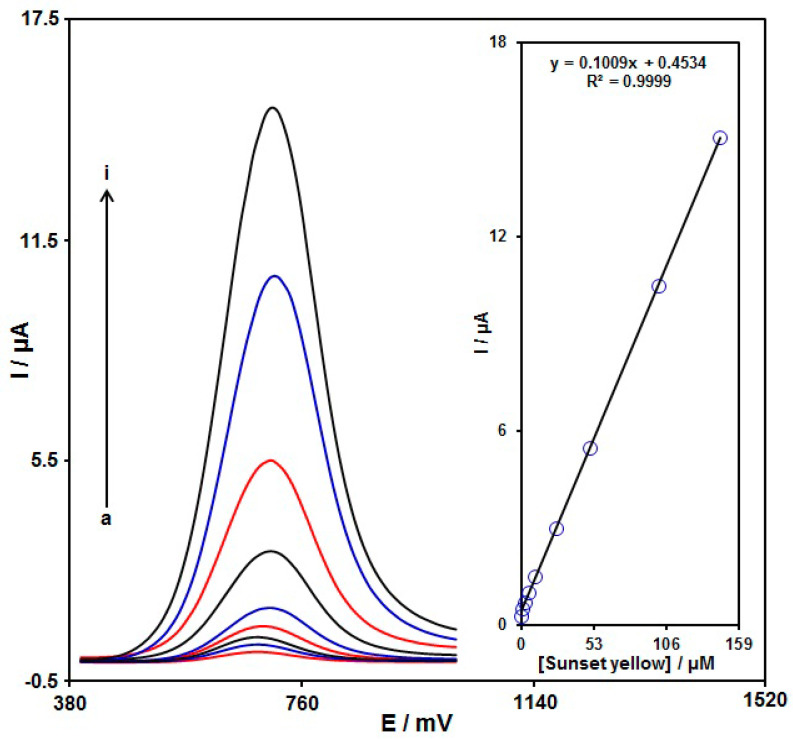
DPVs captured for the oxidation of variable sunset yellow contents on the NiCo_2_O_4_/SPGE (a: 0.02 μM, b: 0.2 μM, c: 2.0 μM, d: 5.0 μM, e: 10.0 μM, f: 25.0 μM, g: 50.0 μM, h: 100.0 μM, and i: 145.0 μM). Inset: Calibration curve of voltammetric response (Ipa) against sunset yellow level.

DPV analysis was done for variable sunset yellow contents to explore the linear dynamic range, LOD, and sensitivity of the NiCo_2_O_4_/SPGE under optimized experimental circumstances (Figure 11). As expected, the elevation in the sunset yellow level enhanced the peak current. Figure 11 (Inset) shows a linear behavior of the oxidation peak currents and variable sunset yellow contents (0.02 μM to 145.0 μM) with the linear regression equation of Ipa (μA) = 0.1009C_sunset yellow_+0.4534 (R^2^ = 0.9999), and the sensitivity of 0.67 μA/(μM.cm^2^). In the equations of LOD = 3S_b_/m and LOQ = 10S_b_/m, the S_b_ stands for the standard deviation of the response for blank solution, and m for the slope from the standard graph. The LOD and LOQ were estimated at 0.008 and 0.024 μM for sunset yellow determination on NiCo_2_O_4_/SPGE.

Table 1 compares the efficiency of the sunset yellow sensor prepared by the hierarchical flower-like NiCo_2_O_4_ nanoplates-modified SPGE and other reported works [1,75,76,77,78,79].

### 3.8. Repeatability, Reproducibility, and Stability

The NiCo_2_O_4_/SPGE was examined for repeatability through the measurement of the response of 50.0 μM sunset yellow on the surface of the same electrode for 10 times. The relative standard deviation (RSD) of 3.4% for the current response of sunset yellow demonstrates the good repeatability of the proposed electrode.

**Figure 12:**
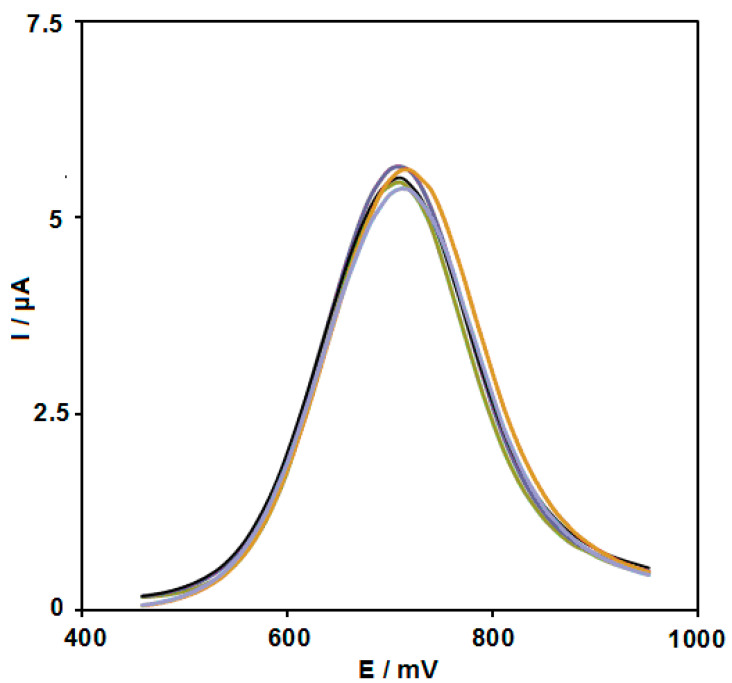
DPVs captured for the oxidation of 50.0 μM sunset yellow contents on the five constructed NiCo_2_O_4_/SPGEs.

To test the reproducibility, five NiCo_2_O_4_/SPGEs produced by the same procedures were applied to measure 50.0 µM sunset yellow under identical circumstances; the obtained RSD of 5.9% meant commendable reproducibility (Figure 12).

To test the NiCo_2_O_4_/SPGE stability, the current responses of 50.0 μM sunset yellow were measured following 14-day storage of the sensor at ambient temperature. The decrease in the peak current of sunset yellow to 95.8% of its original response demonstrated appreciable stability.

### 3.9. Interference Studies

The anti-interference of the NiCo_2_O_4_/SPGE was evaluated by measuring the DPV responses of 50.0 µM sunset yellow with diverse interference matrices. The tolerance limit was defined as the maximum concentration of the interfering substance that caused an approximately ±5% relative error in the determination. It was found that 200-fold Ca^2+^, NH_4_^+^, Mn^2+^, Al^3+^, Fe^3+^, Zn^2+^, Br^−^, Mg^2+^, SO_4_ ^2−^, and CO_3_^2−^ and 20-fold starch, sucrose, uric acid, glucose, vitamin B_6_, vitamin B_2_, dopamine, citric acid, and ascorbic acid had no effect on the determination of the sunset yellow. Hence, the proposed sensor has a good selectivity for sunset yellow.

### 3.10. Analysis of Real Specimens

The practical applicability of NiCo_2_O_4_/SPGE was tested by sensing sunset yellow in orange juice, apple juice, and tap water specimens using the DPV procedure and the standard addition method. The results can be seen in Table 2. The recovery rate was between 96.4% and 104.0%, and all RSD values were ≤3.5%. According to the experimental results, the NiCo_2_O_4_/SPGE sensor possessed a high potential for practical applicability.

## 4. Conclusions

The current attempt was made to construct an effective electrochemical sensing system for detection of sunset yellow via the modification of SPGE with hierarchical flower-like NiCo_2_O_4_ nanoplates (NiCo_2_O_4_/SPGE sensor). The proposed sensor was constructed by a facile and effective drop-casting protocol. Large surface area, high catalytic activity, and great conductivity of flower-like NiCo_2_O_4_ nanoplates provided a good catalytic activity for the NiCo_2_O_4_/SPGE towards the sunset yellow with increased peak current of oxidation and reduced overpotential of oxidation. Electrochemical determinations presented appreciable performance for the modified electrode in the sunset yellow detection at the pH value of 7.0. The as-fabricated NiCo_2_O_4_/SPGE had outstanding analytical response for the sunset yellow detection in a broad linearity range from 0.02 µM to 145.0 µM with a commendable sensitivity of 0.67 μA/(μM.cm^2^) and the LOD as low as 0.008 µM. The practical applicability of the proposed sensor was verified by determining sunset yellow in real matrices, with satisfactory results. The proposed method could be applied to food quality control, and presents a rapid, inexpensive, and environmental-friendly alternative to separation methods.

## Figures and Tables

**Table 1:** Comparison of the efficiency of the NiCo_2_O_4_/SPGE with other reported modified electrodes for sunset yellow determination.

Electrochemical Sensor	Method	Linear Range/μM	LOD/μM	Ref.
Chitosan (CHIT)-graphene (Gr)/glassy carbon electrode (GCE)	CV	0.2–100.0	0.066	[1]
Multi-walled carbon nanotubes (MWCNTs)/GCE	DPV	0.55–7.0	0.12	[75]
Au nanoparticles (NPs)/carbon paste electrode (CPE)	DPV	0.1–2.0	0.03	[76]
Reduced graphene oxide (rGO)-Ni-BTC metal–organic framework (MOF)/Screen-printedcarbon electrode (SPCE)	DPV	0.05–5.0	0.025	[77]
Ionic liquid (IL)-NiFe_2_O_4_-rGO/CPE	DPV	0.05–30.0 and 30.0–500.0	0.03	[78]
Electrochemically reduced oxide (ErGO)/GCE	DPV	0.05–1.0	0.0192	[79]
NiCo_2_O_4_/SPGE	DPV	0.02–145.0	0.008	This Work

**Table 2:** Voltammetric sensing of sunset yellow in real specimens using NiCo_2_O_4_/SPGE. All concentrations are in µM (*n* = 5).

Sample	Spiked	Found	Recovery (%)	R.S.D. (%)
Apple Juice	0	2.5	-	3.2
	2.0	4.6	102.2	2.7
	3.0	5.3	96.4	1.9
	4.0	6.4	98.5	2.9
	5.0	7.6	101.3	3.0
Orange juice	0	3.8	-	2.7
	1.0	4.7	97.9	3.5
	2.0	6.0	103.4	3.1
	3.0	6.9	101.5	1.7
	4.0	7.7	98.7	2.5
Tap Water	0	-	-	-
	5.0	5.1	102.0	2.8
	7.5	7.4	98.7	3.4
	10.0	10.4	104.0	1.8
	12.5	12.4	99.2	2.6

## Data Availability

The data presented in this study are available on request from the corresponding authors.
